# Long term surgical outcomes for infective endocarditis in people who inject drugs: a systematic review and meta-analysis

**DOI:** 10.1186/s12879-019-4558-2

**Published:** 2019-11-08

**Authors:** David Goodman-Meza, Robert E. Weiss, Sebastián Gamboa, Abel Gallegos, Alex A. T. Bui, Matthew B. Goetz, Steven Shoptaw, Raphael J. Landovitz

**Affiliations:** 1 Division of Infectious Diseases, David Geffen School of Medicine at UCLA, 10833 Le Conte Ave (Room 37-121CHS), Los Angeles, CA 90095-1688 USA; 2 Infectious Diseases, VA Greater Los Angeles Healthcare System, Los Angeles, CA USA; 3 Department of Biostatistics, Fielding School of Public Health, UCLA, Los Angeles, CA USA; 4 Universidad Autónoma de Baja California, Tijuana, USA; 5 Medical Imaging Informatics (MII) Group, Department of Radiological Sciences, UCLA, Los Angeles, CA USA; 6 Department of Family Medicine, David Geffen School of Medicine at UCLA, Los Angeles, CA USA; 7 UCLA Center for Clinical AIDS Research & Education, David Geffen School of Medicine, Los Angeles, CA USA

**Keywords:** People who inject drugs, Endocarditis, Surgery, Meta-analysis

## Abstract

**Background:**

In recent years, the number of infective endocarditis (IE) cases associated with injection drug use has increased. Clinical guidelines suggest deferring surgery for IE in people who inject drugs (PWID) due to a concern for worse outcomes in comparison to non-injectors (non-PWID). We performed a systematic review and meta-analysis of long-term outcomes in PWID who underwent cardiac surgery and compared these outcomes to non-PWID.

**Methods:**

We systematically searched for studies reported between 1965 and 2018. We used an algorithm to estimate individual patient data (eIPD) from Kaplan-Meier (KM) curves and combined it with published individual patient data (IPD) to analyze long-term outcomes after cardiac surgery for IE in PWID**.** Our primary outcome was survival. Secondary outcomes were reoperation and mortality at 30-days, one-, five-, and 10-years. Random effects Cox regression was used for estimating survival.

**Results:**

We included 27 studies in the systematic review and 19 provided data (KM or IPD) for the meta-analysis. PWID were younger and more likely to have *S. aureus* than non-PWID. Survival at 30-days, one-, five-, and 10-years was 94.3, 81.0, 62.1, and 56.6% in PWID, respectively; and 96.4, 85.0, 70.3, and 63.4% in non-PWID. PWID had 47% greater hazard of death (HR 1.47, 95% CI, 1.05–2.05) and more than twice the hazard of reoperation (HR 2.37, 95% CI, 1.25–4.50) than non-PWID.

**Conclusion:**

PWID had shorter survival that non-PWID. Implementing evidence-based interventions and testing new modalities are urgently needed to improve outcomes in PWID after cardiac surgery.

## Background

The estimated prevalence of people who inject drugs (PWID) increased worldwide from 2008 to 2017 [1, 2]. PWID are exposed to a wide variety of infectious diseases via injection practices. These infections include HIV, hepatitis B and C viruses, and bacterial and fungal infections. In the United States, the prevalence of people reporting injection drug use in the past year is low (0.3%) [3], yet PWID accounted for 9% of all new HIV cases in 2015 [4] and there has been a more than twofold increase in new HCV cases from 2004 to 2014 [5]. In PWID, however, other bacterial and fungal infectious complications including infective endocarditis (IE) are often overlooked [6].

IE is a severe bacterial or fungal infection of the heart valves, often with blood-stream contamination, with high morbidity and mortality. In PWID, mortality from IE varies from 25 to 35% [7, 8]. IE has increased in PWID, likely related to the recent overall increase in PWID. In the US, the increase in IE has been disproportionate in those who are white, younger, female, and living in rural communities [9, 10]. In comparison to people who do not inject drugs (non-PWID), PWID with IE are typically younger, and have fewer comorbidities or predisposing heart conditions; but are more likely to have more recurrences of IE, be living with HIV, have right-sided valvular disease, and have *Staphylococcus aureus* as the etiologic agent [9–12]. The cost of treating the recurrences of IE of one person who injects drugs was estimated to be $380,000 over a 2-year period [13]; the total costs for treating IE in PWID (*n* = 46) at one hospital was over $8 million in 2012 [14]. Between 2007 to 2017, the costs in North Carolina were estimated to be $78 million [15]. In the US, the total costs for treating bacterial infections in PWID was over $700 million in 2012 [16].

In complicated cases of IE, surgery may be necessary. However, for PWID, surgeons may defer indicated surgery out of concern for worse outcomes largely attributable to reinfection. Indications for surgery in IE classically include signs of severe heart failure, uncontrolled infection (virulent or resistant organisms, persistently positive blood cultures, or perivalvular complications), prevention of embolic events (large vegetations, prior episodes of embolic events), among others [17–19]. Despite a demonstrated mortality benefit for valve replacement surgery in specific clinical scenarios of IE [20–22], US IE guidelines recommend avoiding surgery in PWID if possible due to concerns for reinfection events from continued injecting practices [19]. The ethics of limiting cardiac surgery in PWID has been debated in the literature, and interventions such as signed-contracts agreeing to abstinence, multidisciplinary-team treatment approaches, and even a “three-strike rule” have been proposed [23–30].

Recently, Hall et al. reported a systematic review and meta-analysis that compared survival outcomes at 30 days and in-hospital mortality between PWID and non-PWID [31]. This study found no difference in mortality between the groups early after cardiac surgery. In the present study, our aim was to estimate the long-term survival in PWID post-cardiac surgery for IE, comparing these outcomes to non-PWID. We performed a systematic review and meta-analysis of studies that reported survival after cardiac surgery for patients with IE with the objective to inform clinical practice and future interventions designed to improve outcomes in PWID who are provided surgery for IE.

## Methods

The protocol and methods were registered on PROSPERO, number CRD42018093727 [32]. We report our findings following PRISMA-IPD guidelines [33].

### Literature search

We systematically searched PubMed, Embase, Scopus, and Google Scholar using terms: “injection drug use” or “people who inject drugs” and “infective endocarditis” and “surgery.” The search strategy for each database is provided in Additional file 1: Table S1. We limited our search to human studies between 1965 and 2018. To reduce publication bias, we included both published manuscripts and unpublished conference abstracts. All searches were done between April and May of 2018. We searched the databases without language restriction but only included articles published in English or Spanish. We reviewed the reference list of included articles for other articles that fit our inclusion criteria.

### Selection criteria

We included studies that fulfilled the following criteria: 1) retrospective or prospective design; 2) individuals with infective endocarditis; 3) underwent a surgical cardiac procedure (for example, replaced or repaired a heart valve); and 4) reported baseline or outcomes data for PWID separately from non-PWID. For studies that reported inclusion of PWID but reported their baseline or outcomes data in combination with non-PWID, we contacted the corresponding author for the possibility of separating the data by PWID and non-PWID. We excluded articles that reported data for fewer than 5 PWID.

### Data extraction and quality assessment

Two authors (SG and AG) searched PubMed, Embase, Scopus, and Google Scholar electronic databases for studies that met the inclusion criteria and extracted data independently and in duplicate. We created a standardized protocol for reviewing studies and entered relevant information in an electronic database. Any disagreement in study inclusion or data entry was resolved by consensus of the two authors (SG and AG), or inclusion of a third reviewer (DGM). We extracted data tables for demographic, microbiologic, and valve disease characteristics. For our outcomes, we extracted Kaplan-Meier curves of survival for mortality and reoperation, or tables that reported outcomes for each individual participant. We assessed the quality and reporting of each study using the Newcastle-Ottawa Scale for observational studies. This scale has eight items in three categories: selection, comparability, and outcome. It systematically rates items with one star in the selection and outcomes categories, and up to two stars in the comparability category.

### Variables

We collected data from each study that included location, period of study, age, and gender. We categorized microbiologic pathogens as *Staphylococcus aureus,* coagulase negative staphylococci (CONS), *Streptococci ssp., Enterococci* (we counted Group D Streptococci from older studies with *Enterococci*), gram negative rods (GNR) or others, *Candida*, or culture negative. We extracted data regarding valve-related disease that included the location of the diseased valve (aortic, mitral, tricuspid, pulmonary) and created a separate variable to denote if multiple valves were affected. We also extracted data related to embolic events and prosthetic valve IE. The primary outcome was overall survival defined as the time from cardiac surgery for IE until death, and the secondary outcome was time-to-reoperation survival. We provide estimates for 30-day, 1-year, 5-year, and 10-year survival.

### Statistical analysis

For summary statistics of gender, microbiologic, and valve related characteristics, we summed across studies and calculated percentages. We compared PWID and non-PWID with random-effects meta-analysis. For summary of age, we used random-effects meta-analysis of the mean. To study long-term outcomes, we used a published algorithm by Guyot et al. [34] to estimate individual patient data (eIPD) from digitized Kaplan-Meier curves or used published individual patient data (IPD). The Guyot method was validated by the algorithm’s authors [34] and in a subsequent simulation study [35]. When counts of subjects at risk at regular intervals were absent in the original article, we assumed constant censoring as recommended in the original methodology publication [34]. With the eIPD and IPD, we performed a one-step random effects meta-analysis. In the one-step approach, we pooled all the eIPD and IPD and used a mixed effects Cox Proportional Hazards model to estimate survival in PWID and compare with non-PWID. We adjusted for study and PWID-status by study interaction as random effects of the baseline hazard. We plotted Kaplan-Meier curves broken out by study and constructed a forest plot for the comparative studies. We assessed publication bias visually by funnel plots. All analyses were performed in R (R Development Core Team, Vienna, Austria) using packages *survminer* [36]*, survival* [37]*,* and *meta* [38].

## Results

**Fig. 1:**
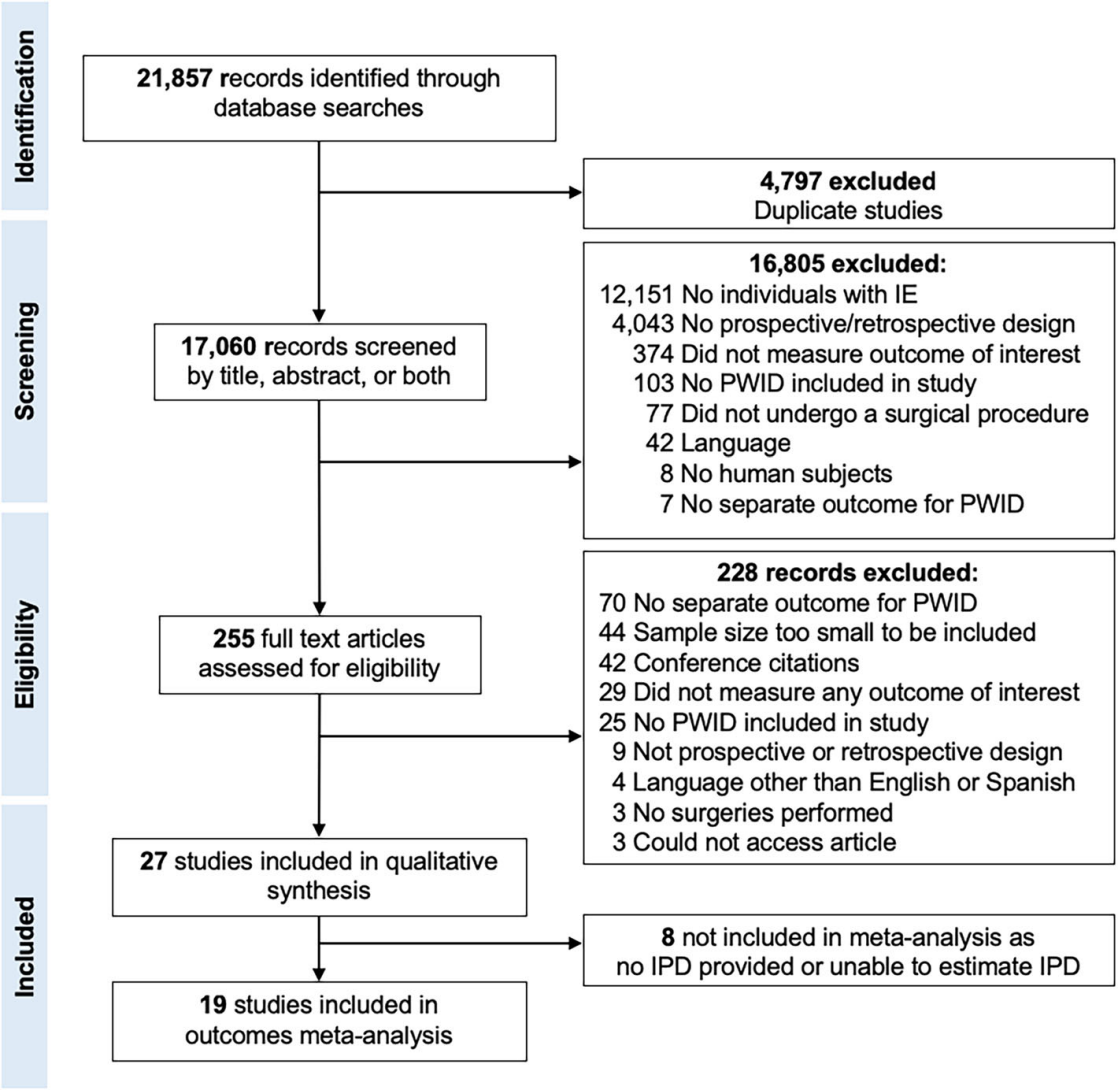
Flow diagram of the study selection process. Abbreviations: *IE* Infective endocarditis, *IPD* Individual participant data, *PWID* People who inject drugs

We identified 21,857 records in the literature search and excluded 4797 duplicates (Fig. 1). Of the remaining 17,060, we excluded 16,805 by reviewing the title, abstract or both. Of 255 remaining records, 228 were excluded by reviewing the body text. We included 27 studies in our qualitative assessment, (Additional file 1: Table S2). All studies were retrospective: 11 compared PWID vs non-PWID, and 16 only reported data for PWID. The majority of studies were from the US (15), followed by Germany (3), Canada (2), Spain (2), Sweden (2), England (1), Italy (1), and Switzerland (1). We recreated eIPD from 13 studies that published Kaplan-Meier curves using the Guyot algorithm and created Kaplan-Meier curves from five studies that published an IPD table (Carrell, Frater, Hubbell, Mammana, and Shetty) and used these data for our primary outcome analysis. Three studies provided survival curves for time-to-reoperation and were used for our secondary outcome analysis. Maximum follow-up ranged from 52 days to 29 years. Data was reported for 926 PWID and 1822 non-PWID. Patients in both PWID and non-PWID groups were majority male, 68.2 and 69.1%, respectively. PWID were younger than non-PWID (mean age, 34.9 years, 95% CI 32.4–37.7, vs. 51.4 years, 95% CI 46.9–56.3, *p* < 0.001). Table 1 describes the characteristics for the included studies.

**Table 1:** Characteristics of included studies

Author	Year Published	Location	Period	PWID	n	Age, mean ± SD	Male, n (%)	Maximum follow-up (years)	Notes
Arbulu [39]	2000	Detroit, USA	1970–1990	Yes	55	NA ± NA	●	29	Kaplan Meier reported for 36 patients.
Asgeirsson [40]	2016	Karolinska, Sweden	2004–2013	Yes	10	38.7 ± 12.75	77 (64)	1	Data reported for surgical and non-surgical cases together.
				No	27	65.6 ± 8.25	96 (77)	1	
Baraki [41]	2013	Hannover, Germany	1996–2012	Yes	9		●		Demographic data reported together. IPD reported in table but survival for survivors not reported.
				No	45	42 ± 16.2	37 (68.5)	5	
Boyd [42]	1977	New York, USA	1970–1975	Yes	14	NA ± NA	●	15	Demographic data reported together. Kaplan Meier not separated by PWID and non-PWID.
				No	19	49 ± 21	19 (57.6)	15	
Carozza [43]	2004	Naples, Italy	1980–2004	Yes	39	32.1 ± 8.1	35 (89.7)	10.5	
				No	85	33.4 ± 8.2	57 (67.1)	10.5	
Carrell [44]	1993	Zurich, Switzerland	1989–1991	Yes	10	23.4 ± 3.2	7 (70)	20	Reconstructed IPD from published table.
Dawood [45]	2015	Baltimore, USA	2002–2012	Yes	56	39 ± 12	23 (41.1)	11	Included 6 patients who were non-PWID
Frater [46, 47]	1989	Bronx, USA	1984–1987	Yes	9	NA ± NA	●	2.75	Reconstructed IPD from published table.
			1977–1980	Yes	10	NA ± NA	●	9	No Kaplan Meier or patient table.
	1990	Bronx, USA	1977–1989	Yes	57	NA ± NA	●	10	Combined previous cohorts
Hubbell [48]	1981	San Francisco, USA	1965–1976	Yes	27	33 ± 12.1	57 (72)	11	Demographic data reported together for surgical and non-surgical. Reconstructed IPD from published table.
Kaiser [49]	2007	St. Louis, USA	1986–2005	Yes	62	39 ± 9	37 (59.7)	19.1	Microbiologic data only for 31 of 62 in PWID.
				No	284	54 ± 15	185 (65.1)	18.8	
Kim [50]	2016	Boston, USA	2002–2014	Yes	78	35.9 ± 9.9	48 (61.5)	10	
				No	358	59.3 ± 14.1	247 (69)	10	
Levitsky [51]	1982	Chicago, USA	1976–1981	Yes	37	34.6 ± NA	31 (83.8)	5	No SD reported. Age range 20–52
Mammana [52]	1983	Chicago, USA	1976–1979	Yes	18	33.7 ± 10.3	●	3	Reconstructed IPD from published table.
Martin-Davila [53]	2005	Madrid, Spain	1985–1999	Yes	26	33 ± NA	●	12	Non-PWID were excluded because they were not separated by surgical and non-surgical patients.
Marks [54]	2015	London, England	1998–2010	Yes	11	27.8 ± 4.8	●	52 days	Data reported together for surgical and non-surgical
Mathew [55]	1995	Chicago, USA	1982–1991	Yes	80	37.7 ± 10	58 (72.5)	7.5	
Mestres [56]	2003	Barcelona, Spain	1985–2002	Yes	21	28.2 ± 6.5	18 (85.7)	10.8	
Nelson [57, 58]	1984	Los Angeles, USA	1972–1982	Yes	27	32.9 ± NA	37 (7110)	10	Demographic data reported together for PWID and non-PWID. Group D streptococcus counted as enterococcus. No SD reported. Age range 9–58
				No	25	38 ± NA		10	
Osterdal [59]	2016	Bergen, Norway	2001–2013	Yes	29	39.7 ± 11.2	27 (93.1)	9.4	
Pfannmueller [60]	2015	Leipzing, Germany	1995–2012	Yes	11	33.5 ± 4.29	8 (72.7)	15	
				No	45	60.1 ± NA	31 (68.9)	15	Used meta-analysis of mean of two non-PWID groups to obtain mean age
Rabkin [61]	2012	Seattle, USA	1999–2010	Yes	64	43.5 ± 10.9	45 (70.3)	6.6	Grouped *S. aureus* and CONS. For analysis, considered as S. aureus.
				No	133	48.4 ± 17.9	97 (72.9)	10.3	
Shetty [62]	2016	Nova Scotia, Canada	2008–2011	Yes	7	33.4 ± 12.1	4 (57.1)	2	Reconstructed IPD from published table.
Shrestha [63]	2015	Cleveland, USA	2007–2012	Yes	41	38 ± 11	26 (63.4)	6	
				No	495	59 ± 14	342 (69.1)	6.4	
Silverman [64]	1984	Chicago, USA	1976–1983	Yes	14	NA ± NA	11 (78.6)	7	
Thalme [10]	2009	Stockholm, Sweden	1994–2000	Yes	60	39 ± 5.9	31 (51.7)	3.3	Combines both surgical and non-surgical. Reason for differences in n (5 PWID and 27 non-PWID)
				No	135	64 ± 19.2	75 (55.6)	9.4	
Weymann [65]	2014	Heidelberg, Germany	1993–2013	Yes	20	35 ± 7.7	13 (65)	16.6	
Ying [66]	2013	Ottawa, Canada	2003–2012	Yes	24	39.4 ± 2.1	17 (70.8)	10	Reported as abstract, never published.
				No	171	59.1 ± 1	127 (74.3)	10	

**Abbreviations:** *CI* Confidence interval, *PWID* People who inject drugs, *non-PWID* people who do not inject drugs, *SD* standard deviation

### Microbiology

In total, 2141 microbiologic pathogens were reported, 738 in PWID from 24 cohorts, and 1403 in non-PWID from nine cohorts. *Staphylococcus aureus* (43.0% vs. 24.7%, *p* = 0.001) was more common in PWID than in non-PWID. *Streptococci* (29.4% vs. 16.7%, p < 0.001)*,* coagulase-negative Staphylococci (13.4% vs. 3.7%, *p* < 0.01), *Enterococci* (12.1% vs. 7.3%, p < 0.01), and culture negative endocarditis (9.6% vs. 5.8%, *p* = 0.01) were more common in non-PWID than in PWID. Summarized microbiologic data are presented in Table 2, and by study microbiologic data are available in Additional file 1: Table S3.

**Table 2:** Microbiologic and valve characteristics of cases undergoing surgery for infective endocarditis

Microbiology								
	Number of pathogens	*Staphylococcus aureus*	CONS	Streptococci	Enterococci	GNR or other	Candida	Culture negative
PWID	738	317 (43)	27 (3.7)	123 (16.7)	54 (7.3)	132 (17.9)	42 (5.7)	43 (5.8)
non-PWID	1403	346 (24.7)	188 (13.4)	412 (29.4)	170 (12.1)	134 (9.6)	19 (1.4)	134 (9.6)
Valve data								
	Number of valves	Mitral	Aortic	Tricuspid	Pulmonary	Multiple	Embolic events	Prosthetic valve
PWID	922	236 (25.6)	366 (39.7)	309 (33.5)	11 (1.2)	145 (15.7)	190 (24.1)	62 (7.9)
non-PWID	1952	719 (36.8)	1037 (53.1)	187 (9.6)	9 (0.5)	246 (12.6)	373 (22.5)	501 (30.2)

**Abbreviations:** *CONS* Coagulase-negative staphylococci, *GNR* Gram negative rods; *non-PWID* People who do not inject drugs, *PWID* People who inject drugs

Numbers denote *n* (%)

Percentages were calculated based on the number of pathogens for the microbiologic variables or number of valves reported for the valve-related variables

### Valve data

The number and type of affected valves were reported by all studies. Two papers (Thalme and Asgerisson) combined valve-related data of those in whom surgery was and was not performed and were not included in aggregated totals. Embolic events were reported for PWID in 10 studies, and non-PWID in six studies. Valve data are presented in Table 2. A total of 2874 valves were included: 922 in PWID and 1952 in non-PWID. In PWID, 39.7% of surgical procedures involved the aortic valve, 33.5% the tricuspid valve, and 25.6% the mitral valve. In non-PWID, 53.1% of surgical procedures involved the aortic valve, 36.8% the mitral valve, and 9.6% the tricuspid valve. Prosthetic valve endocarditis was more common in non-PWID than PWID (30.2 vs. 7.9%, *p* < 0.01). Valve data is broken out by study in Additional file 1: Table S4.

### Outcomes in PWID and non-PWID

**Fig. 2:**
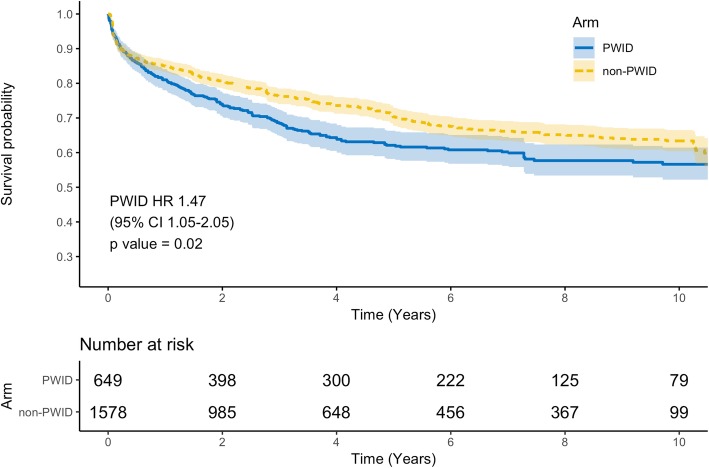
Survival of PWID and non-PWID after cardiac surgery for infective endocarditis. Abbreviations: non-PWID, people who do not inject drugs; PWID, people who inject drugs

**Fig. 3:**
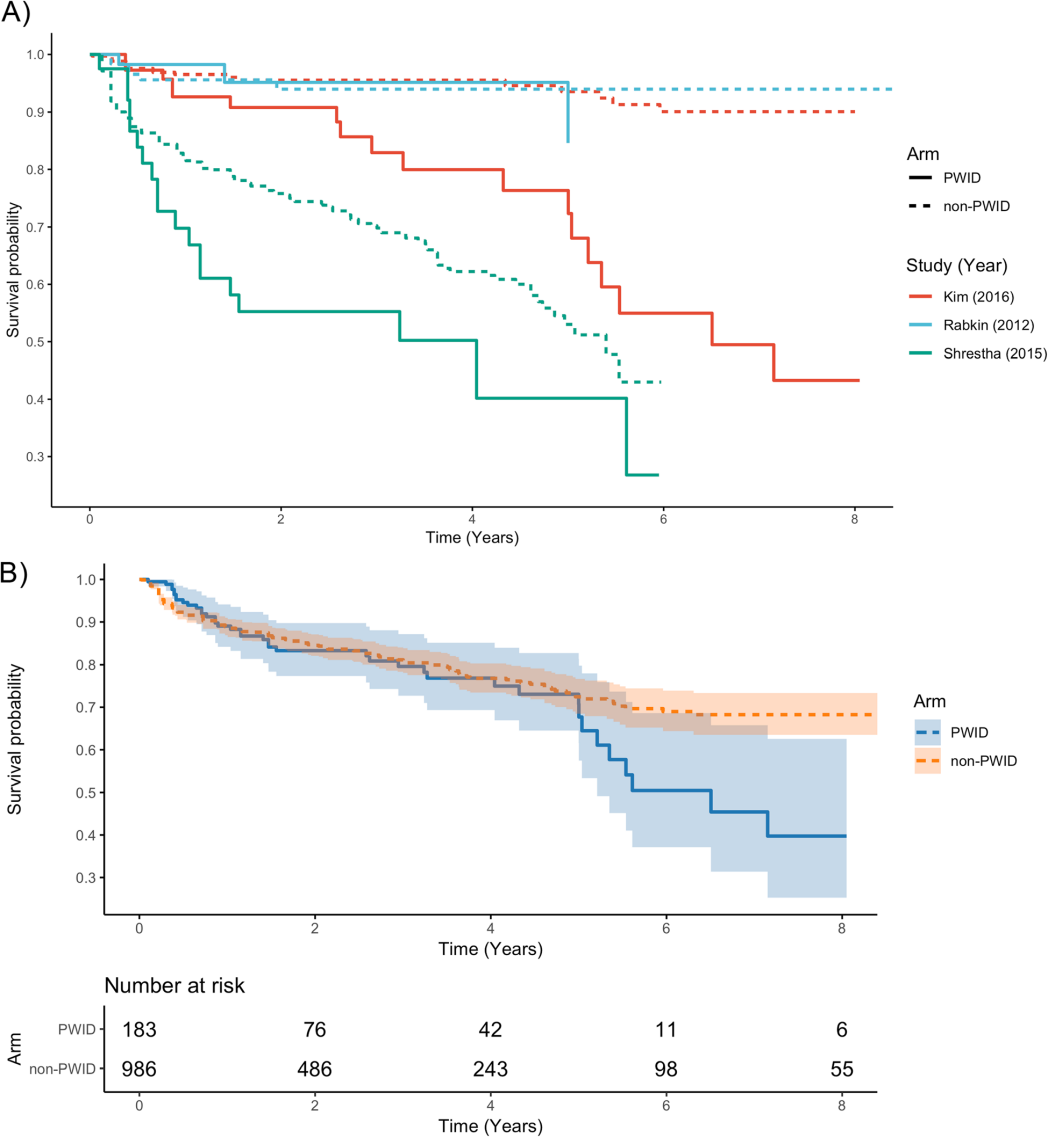
Time-to-reoperation survival of PWID and non-PWID after cardiac surgery for infective endocarditis. Panel A denotes the three included studies. Panel B denotes the aggregate survival curves by arm (PWID vs. non-PWID). Abbreviations: non-PWID, people who do not inject drugs; PWID, people who inject drugs

In one-step random effects meta-analysis of mortality with eIPD from 13 studies and IPD for 5 studies, we included data for 649 PWID and 1578 non-PWID. In PWID, survival was 94.3, 81.0, 62.1 and 56.6% at 30-days, one-, five-, and ten-years, respectively. In non-PWID, survival was 96.4, 85.0, 70.3, and 63.4% at 30-days, one-, five-, and ten-years, respectively (Additional file 1: Table S5). In the mixed effects Cox Proportional Hazards model, the hazard ratio (HR) for PWID was 1.47 (95% CI 1.05–2.05, *p* = 0.02) compared to non-PWID thus non-PWID survive significantly longer than PWID after valvular surgery for IE. Pooled survival curves are presented in Fig. 2. Survival curves by study are presented in Additional file 1: Fig. S1. Survival curves for reoperation were reported by three studies. We estimated IPD for 183 PWID and 986 non-PWID. PWID had a higher hazard of reoperation than non-PWID (HR 2.37, 95% CI, 1.25–4.50, *p* < 0.01). In PWID, median survival to reoperation was 78.1 months (Fig. 3). Funnel plots to visually assess for publication bias were reported in Additional file 1: Fig. S2 and Fig. S3, for the mortality and reoperation outcomes, respectively.

## Discussion

The number of cases of IE in PWID has increased in the US linked to the ongoing opioid epidemic [6, 67]. Consequently, the number of IE cases linked to PWID that require cardiac surgery has also risen, and ethical dilemmas in the decision to offer surgery to PWID have resulted. In this meta-analysis using eIPD and IPD we demonstrated a shorter long-term survival after cardiac surgery for IE for PWID compared to non-PWID. PWID had significantly worse outcomes than non-PWID at 5- and 10-years post-cardiac surgery. At 5 years, PWID had close to 40% mortality. It is notable that on average, PWID who underwent cardiac surgery for IE were approximately 16 years younger than those that are non-PWID. Evidence-based interventions are urgently needed to improve survival in PWID who undergo valve surgery for IE.

There are likely many reasons for the observed mortality and survival disparities. PWID were more commonly infected with *S. aureus*, a highly virulent pathogen associated with worse outcomes in all patients with IE [68]. A higher proportion of PWID infected with *S. aureus* likely led to decreased survival in this group. In parallel to the increase in PWID in the US, invasive methicillin resistant *S. aureus* (MRSA) infections have more than doubled in PWID from 2011 to 2016 and PWID are 16 times more likely to have an invasive MRSA infection than non-PWID [69]. Moreover, MRSA infections have worse outcomes compared to methicillin sensitive *S. aureus* (MSSA) infections [70].

Selection bias may be another reason for worse outcomes in PWID compared to non-PWID. PWID were younger; thus, they would be expected to have fewer medical comorbidities (although this could not be ascertained with the available data). Consequently, PWID would be expected to have longer survival. However, PWID selected for surgery and included in the referenced series may have had more severe cardiac disease, thus biasing the PWID group towards a worse survival. A reason for this bias may be stigma towards PWID, resulting in non-surgical management for milder cases, even when evidence suggests superior outcomes with surgery. Additionally, PWIDs’ perception of stigmatization from healthcare professionals may delay their engagement with appropriate care [71]. Qualitative studies of PWID support the supposition of the influence of stigma, as stigmatizing interactions with hospital staff were found to delay care of injection-related infections [72]. Delays in care seeking may lead to a delay in appropriate non-surgical treatments (such as early antimicrobial administration for antecedent local or bloodstream infections) that could prevent IE and avoid surgery.

Non-treatment of a PWID’s substance use disorder may be an additional reason for our findings. Recurrence of injection drug use after a surgical procedure may have led to increased risk of reoperation in our analysis; however, only a few studies (Asgeirsson, Frater, and Østerdal) reported the proportion of PWID receiving substance use disorder therapy. Despite opioid-type drugs being the most common injection drug in most parts of the world [2] and evidence-based medication-assisted therapies for treating opioid use disorder available since the 1960s [73], few patients actually receive these therapies [7, 74]. In one series of patients with IE, only 10% received substance use disorder therapy [7], and in another series, over half of cases did not have documentation of a discussion regarding addiction treatment [74]. In the only study to our knowledge to test the effect of medication assisted therapy (MAT) in the setting of endocarditis, MAT decreased mortality by close to 70% [75]. More recently, surgical guidelines advocate for a multi-disciplinary approach to the management of IE in PWID but a call for MAT was still lacking [18].

This study has many limitations. The gold standard for performing meta-analysis is obtaining IPD. We used a novel algorithm to obtain eIPD and summarize survival curves. This approach has been validated in the past to produce similar survival estimates to the original study. However, this approach limits us in our ability to analyze survival in the context of more granular covariates of interest (e.g., age, infection with *S. aureus*, right vs. left hearted disease, vegetation size, medical comorbidities, HIV and hepatitis C status, provision of addiction therapy). Surgical technique, intraoperative findings (e.g., abscesses, valve rupture), and post-operative complications may be other variables that may differ between PWID and non-PWID to explain differences in survival but were not assessed as part of this study. Due to the wide range in publication dates of the included articles, cardiac surgery techniques and supportive care likely has improved over the time period but this temporal variation was not assessed. As well, patterns of injection drug use have changed over time and geographic space (i.e., increased fentanyl use or increased injection of stimulants) that may lead to different degrees of severity of IE in PWID. The present results may not be reflective of the current epidemiology of injection drug use. As noted above, selection bias may result from the observational and retrospective nature of the included studies. Another limitation was that we only included studies of non-PWID that had a head-to-head PWID comparison. Studies from other medical centers that only reported outcomes in non-PWID could alter our survival estimates in this group; however, our main objective was the survival of PWID, thus, we chose to not expand our criteria to include studies of non-PWID only.

## Conclusion

In this systematic review and meta-analysis, PWID had shorter survival than non-PWID after cardiac surgery for IE and a higher risk for reoperation compared to non-PWID. With the expected increases in infectious endocarditis in PWID due to the ongoing drug use epidemic in the US and elsewhere, interventions are urgently needed to improve outcomes in this population of young individuals. Interventions that implement substance use disorder treatment and harm-reduction services for patients hospitalized with severe infections and bridge care to the outpatient setting are in need of further study and implementation.

## Supplementary information

**Additional file 1: Table S1.** Search strings for each database. **Table S2.** Newcastle-Ottawa scale for qualitative assessment of included studies (*n* = 27). **Table S3.** Microbiologic characteristics by study of patients undergoing surgery for infective endocarditis. **Table S4.** Valve related characteristics by study of patients undergoing surgery for infective endocarditis. T**able S5.** Survival at 1-month, 1-, 5-, and 10- years. **Fig. S1.** Survival by study of patients that underwent cardiac surgery for infective endocarditis stratified by PWID and non-PWID. **Fig. S2.** Funnel plot of studies comparing mortality in PWID vs. non-PWID after cardiac surgery for infective endocarditis. **Fig. S3.** Funnel plot of studies comparing reoperation in PWID vs. non-PWID after cardiac surgery for infective endocarditis.

## Data Availability

The datasets used and/or analysed during the current study are available from the corresponding author on reasonable request.

## Abbreviations

CI: Confidence interval
CONS: Coagulase negative staphylococci
eIPD: Estimated individual patient data
HR: Hazard ratio
IE: Infective endocarditis
IPD: Individual patient data
MAT: Medication assisted therapy
MRSA: Methicillin resistant *Staphylococcus aureus*
MSSA: Methicillin sensitive *Staphylococcus aureus*
PWID: People who inject drugs
