# Supplementary material for: Long term surgical outcomes for infective endocarditis in people who inject drugs: a systematic review and meta-analysis
Source: BMC Infect Dis. 2019 Nov 8;19:918. doi: 10.1186/s12879-019-4558-2 (PMC6839097; doi:10.1186/s12879-019-4558-2)

| Table S1. Search strings for each database and number of hits. | | |
| --- | --- | --- |
| **Database** | **Search string** |  |
| Embase | ('Cardiac Surgical Procedures'/exp AND 'Endocarditis'/exp AND 'Substance Abuse, Intravenous'/exp OR 'Drug Users'/exp OR "people who inject drugs" OR "injection drug use" OR "intravenous drug abuser" OR PWID OR IVDA OR IVDU OR IDU AND 'Survival'/exp) |  |
| PubMed | "Cardiac Surgical Procedures"[Mesh] AND "Endocarditis"[Mesh] AND "Substance Abuse, Intravenous"[Mesh] OR "Drug Users"[Mesh] OR "people who inject drugs" OR PWID OR "injection drug use" OR "intravenous drug abuser" OR IVDA OR IVDU OR IDU |  |
| Scholar | Valve surgery OR "cardiac surgery" OR "cardiac surgical procedure" AND endocarditis AND "drug user" OR "people who inject drugs" OR PWID OR "injection drug use" OR "intravenous drug abuser" OR IVDA OR IVDU OR IDU AND mortality OR survival OR reoperation |  |
| Scopus | ( ALL ( surg* )  AND  ALL ( endocard* )  AND  ALL ( "Drug Us*"  OR  "person who inject*"  OR  "people who inject*"  OR  "injection drug*"  OR  "Intravenous drug*"  OR  "IDU"  OR  "IVDU"  OR  "IVDA"  OR  "PWID"  OR  "Substance abuse, intravenous" )  AND  ALL ( survival  OR  mortality  OR  reop*  OR  recurr*  OR  outcom*  OR  profile ) ) |  |

Table S2. Newcastle-Ottawa scale for qualitative assessment of included studies (n=27)

|  | **Selection** | | | | **Comparability** | | **Outcome** | | |  |
| --- | --- | --- | --- | --- | --- | --- | --- | --- | --- | --- |
| Criteria | Representativeness of exposed cohort? | Selection of the non-exposed cohort? | Ascertainment of exposure? | Demonstration that outcome of interest was not present at start of study? | Study controls for age? | Study controls for an additional risk factor? | Assessment of outcome? | Was follow-up long enough for outcome to occur? | Adequacy of follow-up of cohorts? | **Total** |
| Acceptable | Truly/somewhat representative of the average IE patient in the community | Drawn from same community as exposed cohort | Secure record/structured interview | All patients alive at start of study | Yes | Yes | Independent blind assessment/record linkage | Follow-up >1 year | <20% subjects lost to follow up |  |
| Arbulu 2000 | * |  |  | * |  |  |  | * | * | 4 |
| Asgeirsson 2016 | * | * | * | * |  | * | * | * | * | 8 |
| Baraki 2013 | * | * | * | * |  |  | * | * | * | 7 |
| Boyd 1977 | * | * |  | * |  |  |  |  | * | 4 |
| Carozza 2004 | * | * | * | * | * | * | * | * | * | 9 |
| Carrell 1993 | * |  |  | * |  |  |  | * | * | 4 |
| Dawood 2015 | * | * | * | * | * | * | * | * | * | 9 |
| Frater 1989 | * | * |  | * |  |  |  | * |  | 4 |
| Hubbell 1981 | * | * | * | * |  |  | * | * | * | 7 |
| Kaiser 2007 | * | * | * | * | * | * | * | * |  | 8 |
| Kim 2016 | * | * | * | * | * | * | * | * |  | 8 |
| Levitsky 1982 | * |  |  | * |  |  |  | * |  | 3 |
| Mammana 1983 | * |  | * | * |  | * | * |  | * | 6 |
| Marks 2014 | * | * | * | * | * | * | * | * | * | 9 |
| Martín-Dávila 2005 | * | * | * | * |  |  | * |  | * | 6 |
| Mathew 1995 | * |  | * | * |  |  |  | * | * | 5 |
| Mestres 2003 | * |  | * | * |  |  | * | * | * | 6 |
| Nelson 1983 | * | * | * | * |  |  |  | * | * | 6 |
| Osterdal 2016 | * |  | * | * |  |  | * | * | * | 6 |
| Pfannmueller 2015 | * | * | * | * |  |  | * | * | * | 7 |
| Rabkin 2012 | * | * | * | * | * | * | * | * | * | 9 |
| Shetty 2016 | * | * | * | * |  |  | * | * | * | 7 |
| Shrestha 2015 | * | * | * | * | * | * | * | * | * | 9 |
| Silverman 1984 | * |  |  | * |  |  |  |  |  | 2 |
| Thalme 2009 | * | * | * | * |  |  | * | * | * | 7 |
| Weymann 2014 | * | * | * | * |  | * | * | * | * | 8 |
| Ying 2013 | * | * | * | * | * | * | * |  |  | 7 |

| Table S3. Microbiologic characteristics by study of patients undergoing surgery for infective endocarditis. | | | | | | | | | | |  |
| --- | --- | --- | --- | --- | --- | --- | --- | --- | --- | --- | --- |
| **Author** | **Year Published** | **PWID** | **n** | **S. aureus** | **Streptococci** | **CONS** | **Enterococci** | **GNR** | **Candida** | **Culture negative** | |
| Arbulu | 2000 | Yes | 74 | 28 (37.8) | 0 (0) | 0 (0) | 0 (0) | 38 (51.4) | 8 (10.8) | 0 (0) | |
| Asgeirsson | 2016 | Yes | 125 | 120 (96.0) | 0 (0) | 0 (0) | 0 (0) | 5 (4.0) | 0 (0) | 0 (0) | |
|  |  | No | 127 | 125 (98.4) | 0 (0) | 0 (0) | 0 (0) | 2 (1.6) | 0 (0) | 0 (0) | |
| Baraki | 2013 | Yes | 14 | 10 (71.4) | 1 (7.1) | 1 (7.1) | 0 (0) | 0 (0) | 0 (0) | 2 (14.3) | |
|  |  | No | 19 | 5 (26.3) | 1 (5.3) | 5 (26.3) | 1 (5.3) | 1 (5.3) | 0 (0) | 6 (31.6) | |
| Boyd | 1977 | Yes | 9 | 5 (55.6) | 1 (11.1) | 0 (0) | 1 (11.1) | 2 (22.2) | 0 (0) | 0 (0) | |
|  |  | No | 46 | 21 (45.7) | 10 (21.7) | 0 (0) | 5 (10.9) | 5 (10.9) | 5 (10.9) | 0 (0) | |
| Carozza | 2004 | Yes | 41 | 20 (48.8) | 9 (22.0) | 6 (14.6) | 1 (2.4) | 2 (4.9) | 0 (0) | 3 (7.3) | |
|  |  | No | 85 | 11 (12.9) | 30 (35.3) | 12 (14.1) | 6 (7.1) | 8 (9.4) | 0 (0) | 18 (21.2) | |
| Carrell | 1993 | Yes | 11 | 7 (63.6) | 2 (18.2) | 0 (0) | 0 (0) | 1 (9.1) | 0 (0) | 0 (0) | |
| Dawood | 2015 | Yes | 56 | 35 (62.5) | 6 (10.7) | 3 (5.4) | 4 (7.1) | 3 (5.4) | 1 (1.8) | 4 (7.1) | |
| Frater | 1989 | Yes | 9 | 9 (100) | 0 (0) | 0 (0) | 0 (0) | 0 (0) | 0 (0) | 0 (0) | |
|  |  | Yes | 10 | 8 (80.0) | 1 (10.0) | 0 (0) | 1 (10) | 0 (0) | 0 (0) | 0 (0) | |
|  | 1990 | Yes |  | - | - | - | - | - | - | - | |
| Nelson | 1984 | Yes | 27 | 5 (18.5) | 5 (18.5) | 0 (0) | 10 (37.0) | 3 (11.1) | 3 (11.1) | 1 (3.7) | |
| Hubbell | 1981 | Yes | 38 | 2 (5.3) | 9 (23.7) | 0 (0) | 0 (0) | 9 (23.7) | 18 (47.4) | 0 (0) | |
| Kaiser | 2007 | Yes | 31 | 18 (58.1) | 10 (32.3) | - | - | 3 (9.7) | - | - | |
|  |  | No |  | - | - | - | - | - | - | - | |
| Kim | 2016 | Yes | 88 | 30 (34.1) | 18 (20.5) | 10 (11.4) | 12 (13.6) | 9 (10.2) | 0 (0) | 9 (10.2) | |
|  |  | No | 381 | 97 (25.5) | 122 (32.0) | 65 (17.1) | 48 (12.6) | 24 (6.3) | 0 (0) | 25 (6.6) | |
| Levitsky | 1982 | Yes | 39 | 5 (12.8) | 5 (12.8) | 0 (0) | 3 (7.7) | 25 (64.1) | 1 (2.6) | 0 (0) | |
| Mammana | 1983 | Yes | 18 | 7 (38.9) | 2 (11.1) | 0 (0) | 0 (0) | 9 (50.0) | 0 (0) | 0 (0) | |
| Marks | 2015 | Yes | 28 | 12 (42.9) | 8 (28.6) | 3 (10.7) | 0 (0) | 0 (0) | 0 (0) | 5 (17.9) | |
| Martin-Davila | 2005 | Yes | 11 | 4 (36.4) | 3 (27.3) | 0 (0) | 2 (18.2) | 0 (0) | 1 (9.1) | 1 (9.1) | |
| Mathew | 1995 | Yes |  | - | - | - | - | - | - | - | |
| Mestres | 2003 | Yes | 27 | 11 (40.7) | 7 (25.9) | 0 (0) | 1 (3.7) | 4 (14.8) | 4 (14.8) | 0 (0) | |
|  |  | No | 28 | 3 (10.7) | 13 (46.4) | 2 (7.1) | 2 (7.1) | 3 (10.7) | 0 (0) | 5 (17.9) | |
| Osterdal | 2016 | Yes | 29 | 15 (51.7) | 4 (13.8) | 0 (0) | 5 (17.2) | 3 (10.3) | 0 (0) | 2 (6.9) | |
| Pfannmueller | 2015 | Yes | 11 | 6 (54.5) | 0 (0) | 1 (9.1) | 1 (9.1) | 0 (0) | 0 (0) | 3 (27.3) | |
|  |  | No | 45 | 18 (40.0) | 7 (15.6) | 9 (20) | 6 (13.3) | 0 (0) | 0 (0) | 5 (11.1) | |
| Rabkin | 2012 | Yes | 64 | 34 (53.1) | 15 (23.4) | - | 4 (6.2) | 3 (4.7) | 0 (0) | 8 (12.5) | |
|  |  | No | 133 | 46 (34.6) | 36 (27.1) | - | 18 (13.5) | 12 (9.0) | 0 (0) | 21 (15.8) | |
| Shetty | 2016 | Yes | 16 | 6 (37.5) | 0 (0) | 0 (0) | 1 (6.2) | 6 (37.5) | 3 (18.8) | 0 (0) | |
| Shrestha | 2015 | Yes | 41 | 24 (58.5) | 5 (12.2) | 1 (2.4) | 3 (7.3) | 5 (12.2) | 2 (4.9) | 1 (2.4) | |
|  |  | No | 495 | 103 (20.8) | 127 (25.7) | 87 (17.6) | 67 (13.5) | 62 (12.5) | 13 (2.6) | 36 (7.3) | |
| Silverman | 1984 | Yes | 14 | 4 (28.6) | 3 (21.4) | 0 (0) | 1 (7.1) | 6 (42.9) | 0 (0) | 0 (0) | |
| Thalme | 2009 | Yes | 60 | 45 (75) | 4 (6.7) | 0 (0) | 7 (11.7) | 3 (5.0) | 0 (0) | 1 (1.7) | |
|  |  | No | 136 | 24 (17.6) | 46 (33.8) | 8 (5.9) | 13 (9.6) | 15 (11.0) | 1 (0.7) | 29 (21.3) | |
| Weymann | 2014 | Yes | 20 | 8 (40) | 4 (20.0) | 2 (10) | 2 (10.0) | 0 (0) | 0 (0) | 4 (20.0) | |
| Ying | 2013 | Yes | 24 | 8 (33.3) | 8 (33.3) | 0 (0) | 4 (16.7) | 1 (4.2) | 2 (8.3) | 1 (4.2) | |
|  |  | No | 171 | 42 (24.6) | 66 (38.6) | 8 (4.7) | 17 (9.9) | 19 (11.1) | 1 (0.6) | 18 (10.5) | |

| Table S4. Valve related characteristics by study of patients undergoing surgery for infective endocarditis. | | | | | | | | | | |
| --- | --- | --- | --- | --- | --- | --- | --- | --- | --- | --- |
| **Author** | **Year Published** | **PWID** | **n** | **Mitral** | **Aortic** | **Tricuspid** | **Pulmonary** | **Multiple** | **Embolic events** | **Prosthetic valve** |
| Arbulu | 2000 | Yes | 57 | 0 (0) | 0 (0) | 55 (96.5) | 2 (3.5) | 2 (3.5) | - | 0 (0) |
| Asgeirsson | 2016 | Yes | 139 | 31 (22.3) | 20 (14.4) | 82 (59.0) | 6 (4.3) | 26 (18.7) | 82 (820) | 7 (70.0) |
|  |  | No | 121 | 56 (46.3) | 59 (48.8) | 5 (4.1) | 1 (0.8) | 7 (5.8) | 29 (107.4) | 21 (77.8) |
| Baraki | 2013 | Yes | 14 | 0 (0) | 0 (0) | 14 (100) | 0 (0) | 0 (0) | - | - |
|  |  | No | 19 | 0 (0) | 0 (0) | 19 (100) | 0 (0) | 0 (0) | - | 0 (0) |
| Boyd | 1977 | Yes | 9 | 1 (11.1) | 5 (55.6) | 3 (33.3) | 0 (0) | 0 (0) | 1 (11.1) | 0 (0) |
|  |  | No | 43 | 12 (27.9) | 28 (65.1) | 3 (7.0) | 0 (0) | 5 (11.6) | 12 (26.7) | 7 (15.6) |
| Carozza | 2004 | Yes | 43 | 8 (18.6) | 23 (53.5) | 12 (27.9) | 0 (0) | 7 (16.3) | 21 (53.8) | - |
|  |  | No | 99 | 55 (55.6) | 37 (37.4) | 7 (7.1) | 0 (0) | 14 (14.1) | 30 (35.3) | - |
| Carrell | 1993 | Yes | 10 | 2 (20.0) | 1 (10) | 7 (70.0) | 0 (0) | 0 (0) | - | - |
| Dawood | 2015 | Yes | 76 | 10 (13.2) | 10 (13.2) | 56 (73.7) | 0 (0) | 20 (26.3) | 36 (64.3) | 1 (1.8) |
| Frater | 1989 | Yes | 11 | 2 (18.2) | 6 (54.5) | 3 (27.3) | 0 (0) | 2 (18.2) | - | 1 (11.1) |
|  |  | Yes | 13 | 5 (38.5) | 5 (38.5) | 3 (23.1) | 0 (0) | 3 (23.1) | - | - |
|  | 1990 | Yes | 71 | 17 (23.9) | 31 (43.7) | 23 (32.4) | 0 (0) | 17 (23.9) | - | 8 (14.0) |
| Nelson | 1984 | Yes | 31 | 7 (22.6) | 20 (64.5) | 4 (12.9) | 0 (0) | 4 (12.9) | - | 2 (7.4) |
| Hubbell | 1981 | Yes | 29 | 6 (20.7) | 21 (72.4) | 2 (6.9) | 0 (0) | 4 (13.8) | - | - |
| Kaiser | 2007 | Yes | 75 | 22 (29.3) | 34 (45.3) | 18 (24.0) | 1 (1.3) | - | - | 9 (14.5) |
|  |  | No | 341 | 152 (44.6) | 157 (46.0) | 31 (9.1) | 1 (0.3) | - | - | 61 (21.5) |
| Kim | 2016 | Yes | 99 | 31 (31.3) | 48 (48.5) | 20 (20.2) | 0 (0) | 21 (21.2) | 36 (46.2) | 17 (21.8) |
|  |  | No | 424 | 144 (34.0) | 262 (61.8) | 18 (4.2) | 0 (0) | 64 (15.1) | 107 (29.9) | 110 (30.7) |
| Levitsky | 1982 | Yes | 41 | 14 (34.1) | 18 (43.9) | 9 (22.0) | 0 (0) | 4 (9.8) | - | 0 (0) |
| Mammana | 1983 | Yes | 20 | 9 (45.0) | 11 (55.0) | 0 (0) | 0 (0) | 2 (10) | - | - |
| Marks | 2015 | Yes | 33 | 9 (27.3) | 16 (48.5) | 8 (24.2) | 0 (0) | 4 (12.1) | - | - |
| Martin-Davila | 2005 | Yes |  | - | - | - | - | - | - | 0 (0) |
| Mathew | 1995 | Yes | 80 | 24 (30.0) | 30 (37.5) | 13 (16.2) | 0 (0) | 13 (16.2) | 15 (18.8) | - |
| Mestres | 2003 | Yes | 27 | 4 (14.8) | 16 (59.3) | 7 (25.9) | 0 (0) | 0 (0) | - | 0 (0) |
|  |  | No | 29 | 7 (24.1) | 19 (65.5) | 3 (10.3) | 0 (0) | 7 (24.1) | - | 3 (12.0) |
| Osterdal | 2016 | Yes | 33 | 4 (12.1) | 24 (72.7) | 4 (12.1) | 1 (3.0) | 7 (21.2) | 17 (58.6) | 1 (3.4) |
| Pfannmueller | 2015 | Yes | 11 | 0 (0) | 0 (0) | 11 (100) | 0 (0) | 0 (0) | 9 (81.8) | 1 (9.1) |
|  |  | No | 45 | 0 (0) | 0 (0) | 45 (100) | 0 (0) | 0 (0) | 20 (44.4) | 4 (8.9) |
| Rabkin | 2012 | Yes | 67 | 27 (40.3) | 31 (46.3) | 6 (9.0) | 3 (4.5) | 11 (16.4) | 14 (21.9) | 14 (21.9) |
|  |  | No | 143 | 43 (30.1) | 91 (63.6) | 6 (4.2) | 3 (2.1) | 18 (12.6) | 24 (18.0) | 42 (31.6) |
| Shetty | 2016 | Yes | 7 | 0 (0) | 0 (0) | 7 (100) | 0 (0) | 0 (0) | 7 (100) | 0 (0) |
| Shrestha | 2015 | Yes | 56 | 19 (33.9) | 14 (25.0) | 22 (39.3) | 1 (1.8) | 14 (25) | 34 (82.9) | 12 (29.3) |
|  |  | No | 598 | 229 (38.3) | 332 (55.5) | 34 (5.7) | 3 (0.5) | 100 (16.7) | 180 (36.4) | 236 (47.7) |
| Silverman | 1984 | Yes | 28 | 13 (46.4) | 10 (35.7) | 5 (17.9) | 0 (0) | 14 (50) | - | 0 (0) |
| Thalme | 2009 | Yes | 64 | 13 (20.3) | 15 (23.4) | 33 (51.6) | 3 (4.7) | 10 (15.6) | - | - |
|  |  | No | 80 | 38 (47.5) | 42 (52.5) | 0 (0) | 0 (0) | 0 (0) | - | - |
| Weymann | 2014 | Yes | 31 | 7 (22.6) | 12 (38.7) | 11 (35.5) | 1 (3.2) | 6 (19.4) | - | 0 (0) |
| Ying | 2013 | Yes | 34 | 12 (35.3) | 11 (32.4) | 9 (26.5) | 2 (5.9) | 7 (20.6) | - | 4 (16.7) |
|  |  | No | 211 | 77 (36.5) | 111 (52.6) | 21 (10.0) | 2 (0.9) | 38 (18) | - | 38 (22.2) |

| Table S5. Survival at 1-month, 1-, 5-, and 10- years   \| **PWID (n=649)** \| \| \| \| **non-PWID (n=1,578)** \| \| \| \|  \| **Random effects Cox model ^1^** \| \| \| \| \| --- \| --- \| --- \| --- \| --- \| --- \| --- \| --- \| --- \| --- \| --- \| --- \| --- \| \|  \| **n** \| **Events** \| **Survival** \| **Mortality** \| **n** \| **Events** \| **Survival** \| **Mortality** \| **HR** \| **Lower** \| **Upper** \| **P value** \| \| 1-month \| 649 \| 37 \| 94.3 \| 5.7 \| 1578 \| 57 \| 96.4 \| 3.6 \| 1.95 \| 0.77 \| 4.95 \| 0.15 \| \| 1-year \| 649 \| 121 \| 81.0 \| 19.0 \| 1578 \| 232 \| 85.0 \| 15.0 \| 1.62 \| 0.95 \| 2.76 \| 0.07 \| \| 5-year \| 649 \| 219 \| 62.1 \| 37.9 \| 1578 \| 391 \| 70.3 \| 29.7 \| 1.66 \| 1.09 \| 2.52 \| 0.02 \| \| 10-year \| 649 \| 234 \| 56.6 \| 43.4 \| 1578 \| 435 \| 63.4 \| 36.6 \| 1.47 \| 1.05 \| 2.05 \| 0.02 \| \| \| Abbreviations: CI, confidence interval; HR, hazard ratio; non-PWID, people who do not inject drugs; PWID, people who inject drugs,  ^1^ Cox Proportional Hazards model adjusted for study and PWID-status by study interaction as random effects of the baseline hazard \| \| --- \| \| \| \| \| \| \| \| \| \| \| \| \| \| |
| --- | --- | --- | --- | --- | --- | --- | --- | --- | --- | --- | --- | --- | --- | --- | --- | --- | --- | --- | --- | --- | --- | --- | --- | --- | --- | --- | --- | --- | --- | --- | --- | --- | --- | --- | --- | --- | --- | --- | --- | --- | --- | --- | --- | --- | --- | --- | --- | --- | --- | --- | --- | --- | --- | --- | --- | --- | --- | --- | --- | --- | --- | --- | --- | --- | --- | --- | --- | --- | --- | --- | --- | --- | --- | --- | --- | --- | --- | --- | --- | --- | --- | --- | --- | --- | --- | --- | --- | --- | --- | --- | --- | --- |


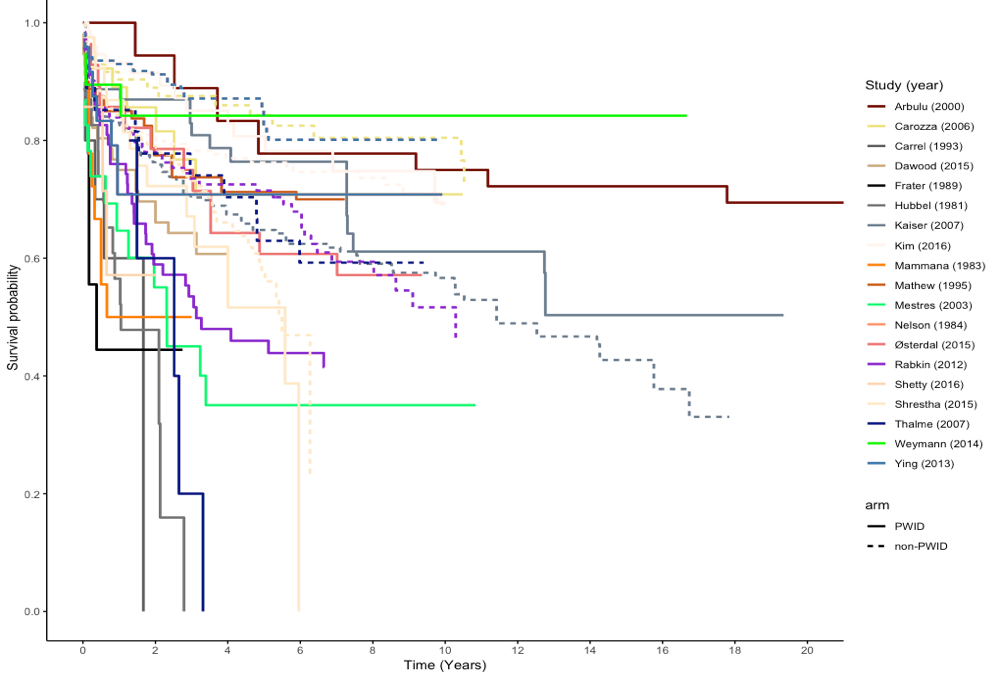
Figure S1. Survival by study of patients that underwent cardiac surgery for infective endocarditis stratified by PWID and non-PWID.

Figure S2. Funnel plot of studies comparing mortality in PWID vs. non-PWID after cardiac surgery for infective endocarditis.


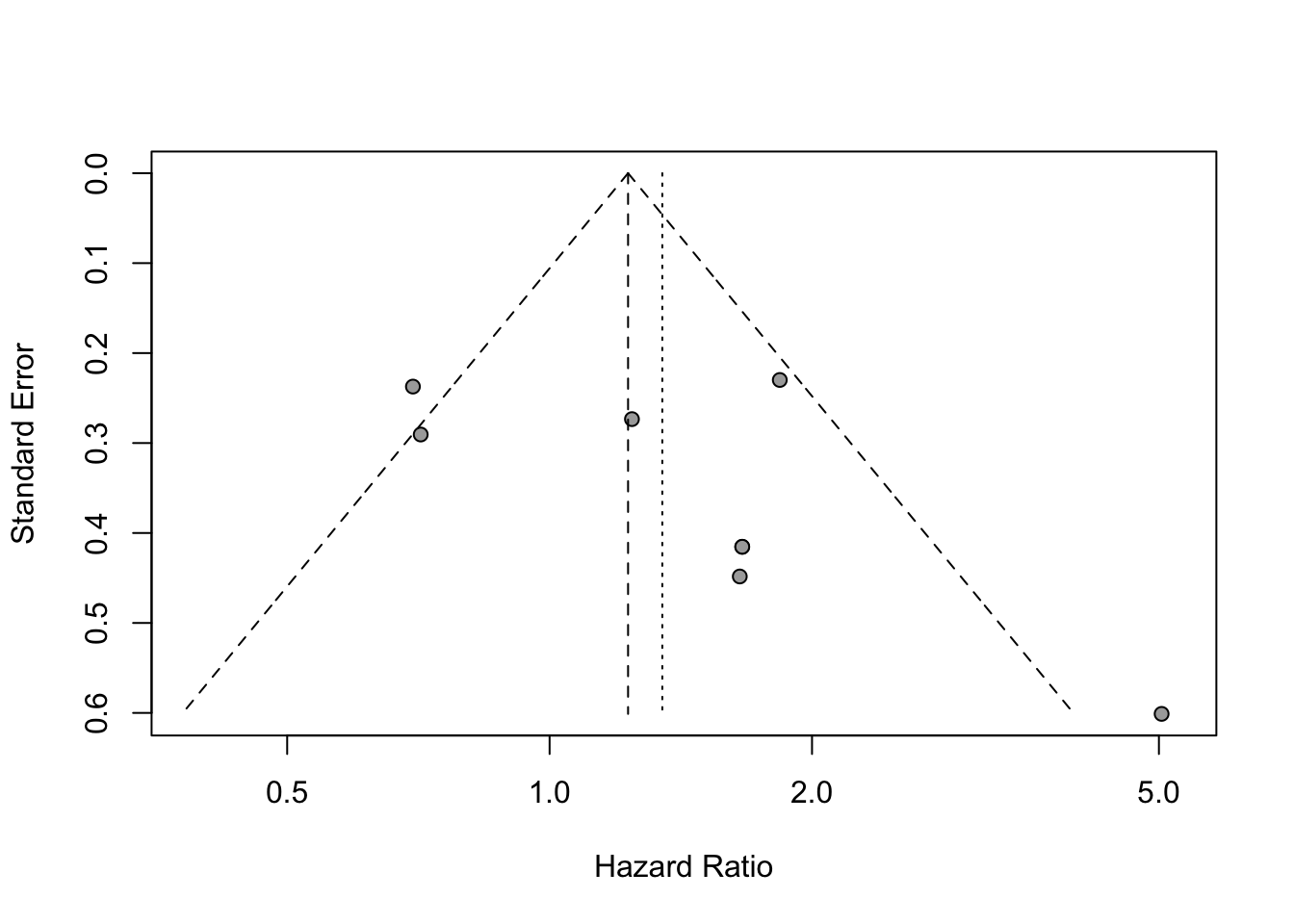


Figure S3. Funnel plot of studies comparing reoperation in PWID vs. non-PWID after cardiac surgery for infective endocarditis.


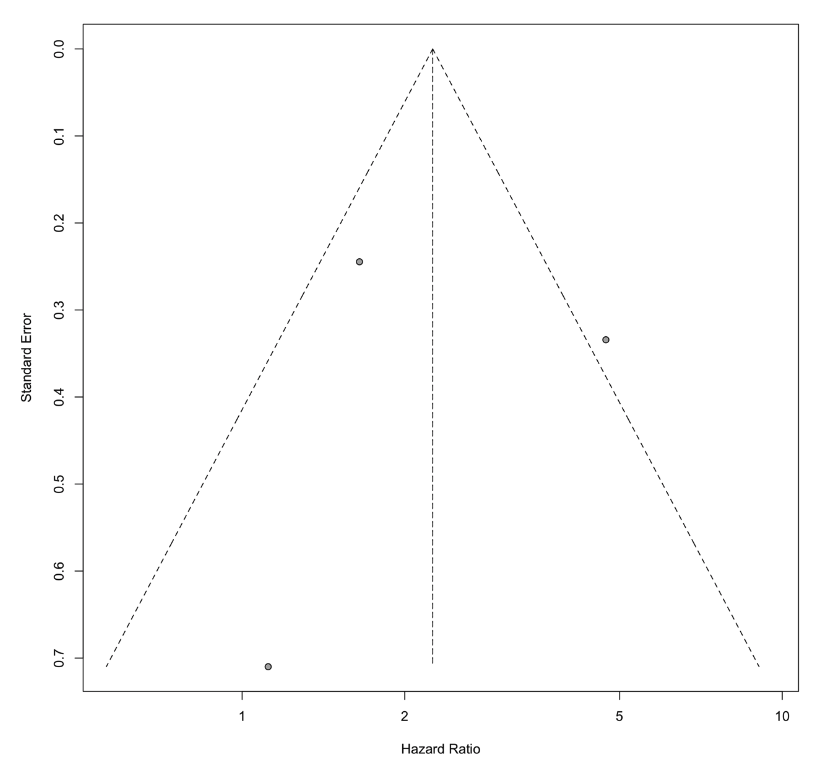

Supplement: Supplementary file 1 — Additional file 1: Table S1. Search strings for each database. Table S2. Newcastle-Ottawa scale for qualitative assessment of included studies (n = 27). Table S3. Microbiologic characteristics by study of patients undergoing surgery for infective endocarditis. Table S4. Valve related characteristics by study of patients undergoing surgery for infective endocarditis. Table S5. Survival at 1-month, 1-, 5-, and 10- years. Fig. S1. Survival by study of patients that underwent cardiac surgery for infective endocarditis stratified by PWID and non-PWID. Fig. S2. Funnel plot of studies comparing mortality in PWID vs. non-PWID after cardiac surgery for infective endocarditis. Fig. S3. Funnel plot of studies comparing reoperation in PWID vs. non-PWID after cardiac surgery for infective endocarditis. [file 12879_2019_4558_MOESM1_ESM.docx]
